# UltraLight VM-UNet: Parallel Vision Mamba significantly reduces parameters for skin lesion segmentation

**DOI:** 10.1016/j.patter.2025.101298

**Published:** 2025-06-26

**Authors:** Renkai Wu, Yinghao Liu, Guochen Ning, Pengchen Liang, Qing Chang

**Affiliations:** 1 Department of Geriatrics, Medical Center on Aging of Ruijin Hospital, Shanghai Jiao Tong University School of Medicine, Shanghai 200025, China; 2 School of Microelectronics, Shanghai University, Shanghai 201800, China; 3 The Innovation Center, Ruijin Hospital, Shanghai Jiao Tong University School of Medicine, Shanghai 200025, China; 4 School of Health Science and Engineering, University of Shanghai for Science and Technology, Shanghai 200093, China; 5 School of Clinical Medicine, Tsinghua University, Beijing 100084, China

**Keywords:** skin lesion segmentation, lightweight model, Mamba

## Abstract

Traditionally, to improve the segmentation performance of models, most approaches prefer to use more complex modules. This is not suitable for the medical field, especially for mobile medical devices, where computationally loaded models are not suitable for real clinical environments due to computational resource constraints. Recently, state-space models, represented by Mamba, have become a strong competitor to traditional convolutional neural networks and transformers. In this paper, we deeply explore the key elements of parameter influence in Mamba and propose an UltraLight Vision Mamba UNet (UltraLight VM-UNet) based on this. Specifically, we propose a method for processing features in parallel Vision Mamba, named the PVM Layer, which achieves competitive performance with the lowest computational complexity while keeping the overall number of processing channels constant. We conducted segmentation experiments on three public datasets of skin lesions and showed that UltraLight VM-UNet exhibits competitive performance with only 0.049M parameters and 0.060 GFLOPs.

## Highlights

• Mamba was analyzed in depth for its parameter key influences
• A new framework and theoretical analysis for lightweight model of medical scenarios
• Parallel Vision Mamba (or Mamba) is a winner for lightweight models
• Provides an UltraLight Vision Mamba UNet for mobile skin lesion segmentation

## The bigger picture

Previously, medical image segmentation models were often constrained by computational resources and memory, limiting their application in mobile medical devices or resource-constrained environments. In contrast, the UltraLight VM-UNet model proposed in this study, with its low parameter count and computational complexity, is able to reduce the requirements on device resources without sacrificing performance. This means that it can conveniently assist doctors in diagnosing diseases in more scenarios, such as on medical devices in remote areas or in emergency medical rescue for fast processing of medical image data. In the long run, the ambition of this research is to promote the popularization and efficiency of medical image segmentation technology. It is hoped that accurate medical image diagnosis will no longer be limited to high-end equipment in large hospitals, but can be extended to all kinds of primary healthcare organizations, and even portable medical equipment, so that more patients can be diagnosed in a timely and accurate manner. Moreover, the lightweight module proposed in this study is plug-and-play, which can simply replace the redundant modules in other technology models, reducing the requirements on equipment resources while maintaining excellent performance. This will promote the rapid development of lightweight medical image processing, improve the overall quality and popularity of medical services, and bring positive social impacts.

## Graphical abstract

**Figure:**
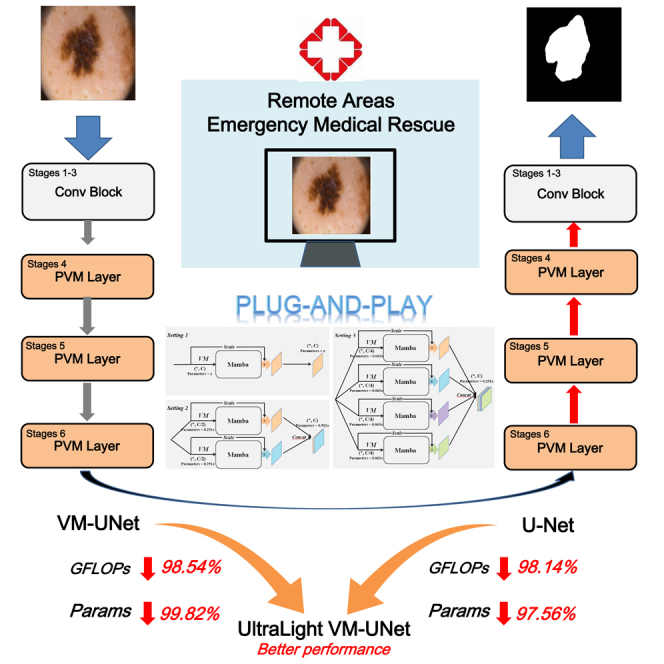

## Teaser

In the field of computer-aided medical diagnosis, medical image segmentation techniques (e.g., skin lesion segmentation) have been a key research hotspot. In this paper, the authors propose the UltraLight VM-UNet model, which operates efficiently in resource-constrained environments while guaranteeing high segmentation performance through an innovative PVM Layer. These results have implications for enhancing the diagnostic capabilities of mobile medical devices and promoting the widespread application of medical image segmentation technology.

## Introduction

With the rapid advancement of computer technology and hardware capabilities, computer-aided diagnosis has seen widespread adoption in the medical domain, with medical image segmentation serving as a critical component. Modern segmentation methods are predominantly powered by deep learning networks, particularly those based on convolutional neural networks and transformers. Convolutional architectures excel at extracting local features, yet they struggle to capture long-range dependencies effectively.^1^^,^^2^^,^^3^ To address this limitation, previous works^4^^,^^5^ have explored the use of large convolutional kernels, aiming to extend the receptive field and thus improve the modeling of distant spatial relationships. On the other hand, transformer-based architectures have recently garnered significant attention in medical image analysis.^6^^,^^7^ Their self-attention mechanism inherently facilitates global context modeling by operating over sequences of image patches. However, this advantage comes at the cost of increased computational complexity, as the self-attention operation scales quadratically with respect to the input image size.

Moreover, to enhance the accuracy of computer-aided diagnosis, many existing approaches tend to increase the number of model parameters to boost predictive performance.^8^^,^^9^ However, such strategies are often impractical in real-world clinical settings, where computational power and memory resources are inherently limited. In the context of mobile health applications, models must meet strict requirements for low parameter counts and minimal memory consumption.^10^ Consequently, there is a pressing need for algorithmic models that can deliver strong performance while maintaining low computational complexity—making them well suited for deployment on future mobile medical devices.

Recently, state-space models (SSMs) have shown linear complexity in terms of input size and memory occupation,^11^^,^^12^ which makes them key to lightweight model foundations. In addition, SSMs excel at capturing remote dependencies, which can critically address the problem of convolution for extracting information over long distances. In Gu and Dao,^13^ time-varying parameters were introduced into an SSM to obtain Mamba, and it was demonstrated that Mamba is able to process textual information with lower parameters than transformers. On the vision side, the introduction of Vision Mamba (VM)^12^ has once again furthered people’s understanding of Mamba, which saves 86.8% of memory when reasoning about images of 1,248×1,248 size without the need for an attentional mechanism. With the outstanding work of the researchers mentioned above, we are more confident that Mamba will occupy a major position in the future as a basic building block for lightweight models.

**Figure 1:**
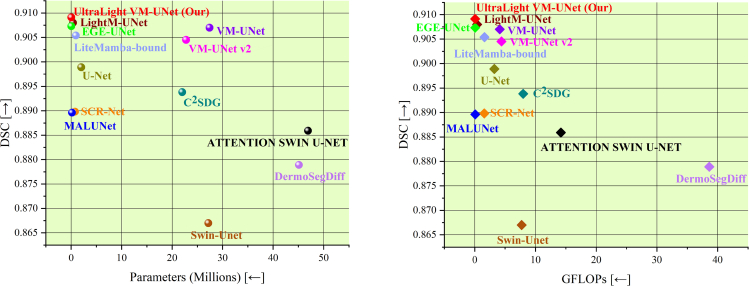
Visualization of the comparison results for the ISIC2017 dataset The *X* axis corresponds to parameters and GFLOPs, the fewer the better. The *Y* axis corresponds to segmentation performance (DSC), the higher the better.

**Figure 2:**
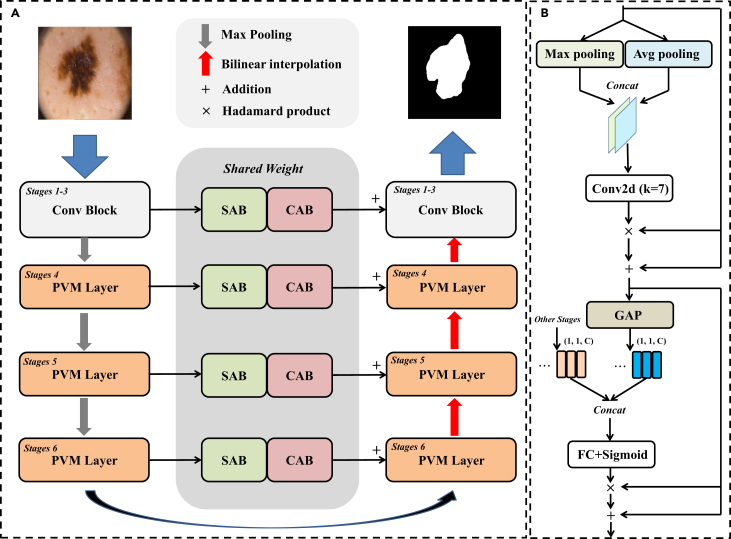
The proposed UltraLight Vision Mamba UNet (UltraLight VM-UNet) model architecture (A) The main part of the UltraLight VM-UNet. (B) The skip-connection part of the UltraLight VM-UNet.

In this paper, we propose a lightweight model based on VM. We deeply explore the critical memory footprint of Mamba and the performance trade-offs, and propose an UltraLight Vision Mamba UNet (UltraLight VM-UNet). The proposed UltraLight VM-UNet represents an ultra-lightweight VM model with only 0.049M parameters and 0.060 GFLOPs, demonstrating highly competitive performance across three skin lesion segmentation tasks (Figure 1). Specifically, we delve into the keys affecting the computational complexity in Mamba, and conclude that the number of channels is a key factor in the explosive memory occupation for Mamba computation. We build on this finding of ours by proposing a parallel Vision Mamba (PVM) approach for processing deep features, named the PVM Layer, which simultaneously keeps the overall processing channel count constant. The proposed PVM Layer achieves excellent performance with surprisingly low parameters. In addition, the deep feature extraction of the proposed UltraLight VM-UNet that we implement using only the PVM Layer containing Mamba, as shown in Figure 2. In the methods section, we present the details of the proposed UltraLight VM-UNet as well as the key factors of the parameter effects in Mamba and the performance balancing approach.

Although similar parallel connection of modules has been mentioned in previous studies,^14^^,^^15^^,^^16^^,^^17^ the impact of using parallel connection in Mamba is still unknown. Does parallel connection lead to a significant performance degradation of Mamba when utilizing the SSM selection mechanism? This is because parallel connections lead to a reduction in the number of feature channels learned per SSM. In this paper, we give the answer in detail. PVM or Mamba not only remain competitive in terms of performance, but achieve a significant reduction in parameters and computational complexity.

Our contributions and findings can be summarized as follows:

(1) An UltraLight Vision Mamba UNet (UltraLight VM-UNet) is proposed for skin lesion segmentation with parameters of only 0.049M and GFLOPs of only 0.060.
(2) A PVM method for processing deep features, named the PVM Layer, is proposed, which achieves excellent performance with the lowest computational complexity while keeping the overall number of processing channels constant. This can be generalized to the parallel connection of any Mamba variant.
(3) We provide an in-depth analysis of the key factors influencing the parameters of Mamba, and provide a theoretical basis for Mamba to become a mainstream module for lightweight models in the future.
(4) The proposed UltraLight VM-UNet parameters are 99.82% lower than the traditional pure Vision Mamba UNet model (VM-UNet) and 87.84% lower than the parameters of the current lightest Vision Mamba UNet model (LightM-UNet). In addition, the UltraLight VM-UNet maintains strong performance competitiveness in all three publicly available skin lesion segmentation datasets.

## Results

### Architecture overview

The proposed UltraLight VM-UNet is shown in Figure 2. UltraLight VM-UNet has a total of 6 layers in a structure consisting of a U-shaped structure (encoder, decoder, and skip-connection path). The number of channels in the 6-layer structure is set to [8,16,24,32,48,64]. The extraction of shallow features in the first 3 layers is composed using a convolution module (Conv Block), where each layer includes a standard convolution with a 3×3 kernel and a maximum pooling operation. The deeper features from layer 4 to layer 6 are our core part, where each layer consists of our proposed PVM Layer. The decoder part maintains the same setup as the encoder. The skip-connection path utilize the Channel Attention Bridge (CAB) module and the Spatial Attention Bridge (SAB) module for multilevel and multiscale information fusion.^18^

### Mamba parameter impact analysis

**Figure 3:**
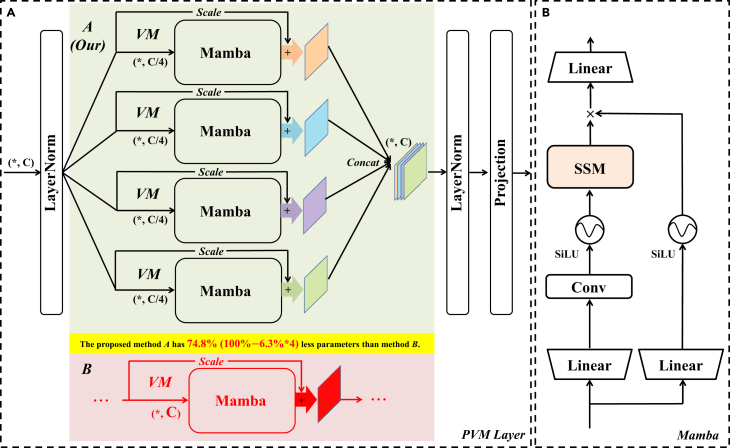
The proposed PVM Layer architecture (A) The main part of the PVM Layer. VM is composed by Mamba combined with residual connection and adjustment factor. (B) Mamba composition structure.

The VM for PVM Layer is mainly composed using Mamba combined with residual connections and adjustment factors (Figure 3A), which allows traditional Mamba to improve the capture of remote spatial relations without introducing additional parameters and computational complexity.^19^ This has a better improvement in the performance of Mamba in visual tasks while keeping the parameter and computational complexity low.

Among SSM-based Mamba, the number of channels, the size of the SSM state dimension, the size of the internal 1D convolutional kernel, the projection dilation multiplier, and the rank of the step size all affect the parameters. And in this, the impact of the channel number is explosive, and its main influence is from the following multiple directions:

First, d_inner of the Mamba internal extended projection channel is determined by the product of the projection expansion multiplier and the number of input channels. This can be specifically expressed by the following equation:

(Equation 1) d_inner=expand∗d_model

where d_inner is the internal expansion projection channel, expand is the projection expansion multiplier (fixed at 2 by default), and d_model is the number of input channels. We can conclude that d_inner will double up as the number of channels (d_model) per layer in the model increases.

Second, the parameters of the input projection layer (the same input linear layer is used for both branches) and the output projection layer within Mamba will be directly related to the number of input channels. The input projection layer and output projection layer operate as follows:

(Equation 2) in_proj:nn.Linear(d_model,d_inner∗2,bias=False)

(Equation 3) out_proj:nn.Linear(d_inner,d_model,bias=False)

where the input projection (in_proj) layer parameter is d_model∗d_inner∗2, and the output projection layer (out_proj) parameter is d_inner∗d_model. We can conclude that the number of input channels, d_model, is the key element controlling the parameter, where the internal extended projection channel, d_inner, is also controlled by d_model.

Further, the intermediate linear projection layers of SSM are also key to the influence of the parameters. The details are as follows:

(Equation 4) x_proj=nn.Linear(d_inner,(dt_rank+d_state∗2),bias=False)

(Equation 5) dt_proj=nn.Linear(dt_rank,d_inner,bias=True)

where dt_rank is the rank of the step (dt_rank=d_model/16), d_state is the size of the state dimension (fixed to 16), where the parameters can be derived as d_inner∗(dt_rank+d_state∗2). dt_proj is a linear projection layer for step size, with parameters (dt_rank∗d_inner)+d_inner, mainly used for linear projection for step size (dt). So, we can conclude that all parameters are still mainly controlled by the number of input channels d_model.

In addition, the internal convolution (nn.Conv1d(d_inner,d_inner,d_conv,bias=True)) also provides parametric influence with d_inner∗d_inner∗d_conv+d_inner. In this paper, d_conv is fixed to 4, so the convolution provides a parameter of $4*d\_inner^{2}+d\_inner$, which is also controlled by the d_model.

Also, the logarithmic form parameters (A_logs) of the transfer matrix A in the SSM module are an important influencing element. A_logs is a parameter matrix of the shape (d_inner, d_state), so its parameters can be derived as d_inner∗d_state. In addition, the trainable vector parameter (D) of the process computed within the SSM contains the d_inner parameter, which is used to selectively integrate the SSM state outputs with the original input signals, thus enhancing the model’s expressiveness and training stability.

In summary, assuming that the original channel number is 1,024, keeping other parameters unchanged, and when the channel number is reduced to one-fourth of the original (channel number 256), the original total parameters can be calculated from the above parameter formula to get the original total parameters reduced from 23,435,264 to 1,484,288. The parameter explosion is reduced by 93.7%, which further confirms that the number of channels has a very critical impact on Mamba parameters.

Building upon the in-depth analysis of the key factors influencing parameter efficiency in Mamba, we propose a PVM Layer for feature processing. This design achieves outstanding performance with minimal calculated load, while maintaining a constant total number of processing channels. The architectural details and implementation of the PVM Layer are presented in the following section.

### Mamba variant (SS2D) parameter impact analysis

The 2D Selective Scan (SS2D) have been developed based on SSM which are more suitable for visual tasks, and SS2D are usually embedded in a Visual State Space (VSS) Block.^11^ The VSS Block consists of two main branches, the first one mainly consists of a linear layer and SiLU activation function.^20^ The second branch is mainly composed of linear layers, convolution, SiLU activation function, SS2D, and LayerNorm. Finally, the two branches merge the outputs by element-by-element multiplication.

The components of SS2D include scan expansion operation, S6 block feature extraction, and scan merge operation. The sequence is first expanded in four directions from top-left to bottom-right, bottom-right to top-left, top-right to bottom-left, and bottom-left to top-right by scan expansion operation. Subsequently, the extracted features are passed through the S6 block^13^ for deep feature refinement. A scan merge operation is then employed to reconstruct the spatial resolution to match that of the original input image.

In the VSS Block, the number of input channels, the size of the state dimension of the S6 block, the size of the internal convolution kernel, the projection dilation multiplier, and the rank of the projection matrix all affect the parameters. Among them, the influence of the number of input channels is explosive, and its influence mainly comes from the following aspects.

First, the d_inner of the VSS Block internal extended projection channel is determined by the product of the projection expansion multiplier and the number of input channels. This can be specifically expressed by the following equation:

(Equation 6) d_inner=expand∗d_model

where d_inner is the internal expansion projection channel, expand is the projection expansion multiplier (fixed at 2 by default), and d_model is the number of input channels. We can see that d_inner will rise exponentially as the number of channels per layer in the model increases dramatically.

Second, the parameters of the input projection layer (the same input linear layer is used for both branches) and output projection layer within VSS Block will be directly related to the number of input channels. The input projection layer and output projection layer operate as follows:

(Equation 7) in_proj:nn.Linear(d_model,d_inner∗2,bias=False)

(Equation 8) out_proj:nn.Linear(d_inner,d_model,bias=False)

where the input projection (in_proj) layer parameter is (d_model∗d_inner∗2), and the output projection layer (out_proj) parameter is (d_inner∗d_model). In addition, the output section has a layer normalization operation (LayerNorm) with parameter (d_inner+d_inner). We can see that the number of input channels, d_model, is the key element controlling the parameter, where the internal extended projection channel, d_inner, is also controlled by d_model.

Further, the linear projection layers in the S6 block of SS2D are also key to the parameter effects. Each linear projection layer is specified as follows:

(Equation 9) x_proj=nn.Linear(d_inner,(dt_rank+d_state∗2),bias=False)

(Equation 10) dt_proj=nn.Linear(dt_rank,d_inner,bias=True)

where dt_rank is the rank of the projection matrix (dt_rank=d_model/16), d_state is the size of the S6 block state dimension (fixed to 16), and the parameters for each linear projection layer are (d_inner∗(dt_rank+d_state∗2)). However, there are 4 linear projection layers in total, so the total parameters are 4∗(d_inner∗(dt_rank+d_state∗2)). In addition, dt_proj is a linear projection layer for step size with parameter dt_rank∗d_inner+d_inner, which is mainly used for linear projection for step size (dt). dt_proj also has 4 layers, with a total parameter of 4∗(dt_rank∗d_inner+d_inner). So, from the above, we can know that all parameters are still mainly controlled by the number of input channels d_model.

In addition, the internal convolution (nn.Conv2d(d_inner,d_inner,d_conv,bias=True)) also provides parametric influence with d_inner∗d_inner∗d_conv∗d_conv+d_inner. In this paper, d_conv is fixed to 3, so the convolution provides a parameter of $3^{2}*d\_inner^{2}+d\_inner$, which is also controlled by the d_model.

Also, the A_logs of the parameter matrix controlling the attention weights of the different states of the S6 block in the SS2D module is an important influencing element. A_logs is a parameter matrix of the shape (K∗d_inner,d_state), and K is a hyperparameter that is usually fixed to 4. Therefore, the parameter A_logs can be derived as d_inner∗d_state∗4. In addition, the trainable vector parameter (Ds) of the process computed within the SS2D contains the 4∗d_inner parameter, which is used to selectively integrate the SS2D state outputs with the original input signals, thereby enhancing the model’s expressiveness and training stability.

In summary, assuming that the original number of input channels is 1,024, keeping the other parameters unchanged, and reducing the number of channels to a quarter of the original (the number of input channels becomes 256), the original total parameters can be calculated by the above parameter formulae from 45,504,512 to 2,921,984. The parameter explosion reduces the number of channels by 93.6%, which further confirms that the number of input channels has a very critical impact on the VSS Block parameters.

### PVM Layer

As analyzed in the previous subsection, the number of input channels has an explosive effect on the parameters of Mamba. As shown in Figure 3A, we propose the PVM Layer for processing deep features. Specifically, a feature X with channel number C first passes through a LayerNorm layer and then is divided into $Y_{1}^{C/4}$, $Y_{2}^{C/4}$, $Y_{3}^{C/4}$, and $Y_{4}^{C/4}$ features each with channel number C/4. After that, each of the features is then fed into Mamba, and then the output is subjected to residual concatenation and adjustment factor for optimizing the remote spatial information acquisition capability.^19^ Finally, the four features are combined into the feature $X_{out}$ with channel number C by concat operation, and then output by LayerNorm and Projection operation, respectively. The specific operations can be expressed by the following equations:

(Equation 11) $$Y_{1}^{C/4},Y_{2}^{C/4},Y_{3}^{C/4},Y_{4}^{C/4}=Sp\left[\mathrm{LN}\left(X_{in}^{C}\right)\right]$$

(Equation 12) $$VM\_Y_{i}^{C/4}=Mamba\left(Y_{i}^{C/4}\right)+\theta \cdot Y_{i}^{C/4}\;i=1,2,3,4$$

(Equation 13) $$X_{out}=Cat\left(VM_{-}Y_{1}^{C/4},VM_{-}Y_{2}^{C/4},VM_{-}Y_{3}^{C/4},VM_{-}Y_{4}^{C/4}\right)$$

(Equation 14) $$Out=Pro\left[\mathrm{LN}\left(X_{out}\right)\right]$$

where LN is the LayerNorm, Sp is the Split operation, Mamba is the Mamba operation, θ is the adjustment factor for the residual connection, Cat is the concat operation, and Pro is the Projection operation. From Equation 12, we used PVM processing features, while ensuring that the total number of channels processed remains constant, maintaining high accuracy while maximizing parameter reduction. As shown in Figure 3A for methods A and B, again assuming a channel count size of 1,024, each VM in method A reduces the parameters by 93.7%, and it contains 4 such operations; when summed up, the comparison method B parameters are reduced by 74.8% overall. Through our proposed PVM operation, the parameter reduction is maximized while maintaining strong performance competitiveness.

### Quantitative and qualitative analysis

To validate the performance of the proposed UltraLight VM-UNet under the 0.049M parameter, we conducted comparison experiments with several state-of-the-art lightweight and classical medical image segmentation models. Specifically, they include U-Net,^21^ SCR-Net,^22^ Swin-Unet,^23^ ATTENTION SWIN U-NET,^8^ $C^{2}$ SDG,^24^ VM-UNet,^25^ VM-UNet v2,^26^ MALUNet,^18^ LightM-UNet,^19^ EGE-UNet,^10^ DermoSegDiff,^27^ and LiteMamba-Bound.^28^

**Figure 4:**
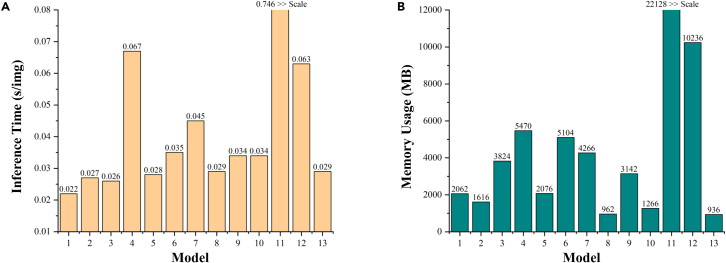
Comparison of inference time and memory usage between the proposed and compared methods (A) Comparison of inference time for different models. (B) Comparison of memory usage for different models. Numbers 1–13 refer to U-Net, SCR-Net, Swin-Unet, ATTENTION SWIN U-Net, $C^{2}$ SDG, VM-UNet, VM-UNet v2, MALUNet, LightM-UNet, EGE-UNet, DermoSegDiff, LiteMamba-Bound, and UltraLight VM-UNet (Our).

Table 1 show the experimental results on the ISIC2017, ISIC2018, and ${\text{PH}}^{2}$ datasets, respectively. As shown in the table, the parameters of our model are 99.82% lower than those of the traditional pure VM-UNet and 87.84% lower than those of the current LightM-UNet. In addition, the GFLOPs of our model are 98.54% lower than VM-UNet and 84.65% lower than LightM-UNet. With such a large reduction in parameters and GFLOPs, the performance of our model still maintains excellent and highly competitive performance. In addition, MALUNet is a lightweight model proposed based on convolution, and although it has lower parameters and GFOLPs than VM-UNet and LightM-UNet, the parameters and GFOLPs of our model are still 72.0% and 27.71% lower than them, respectively. In particular, the performance of MALUNet, the proposed lightweight model based on convolution, is much lower than that of the Mamba-based model, which reflects that it is difficult for the convolution-based lightweight model to balance the relationship between performance and computational complexity. In addition, for a comprehensive analysis of the results, the proposed UltraLight VM-UNet also exhibits slightly lower values for some metrics compared with other models in the ISIC2017 and ISIC2018 datasets. For example, the specificity (SP) of UltraLight VM-UNet is lower than that of other comparison models in the ISIC2017 and ISIC2018 datasets. This is due to the fact that the proposed UltraLight VM-UNet uses multiple PVM focusing on the target region to maintain the ultra-lightweight architecture, but this also affects the model’s ability in recognizing the background, which leads to a slightly lower SP index. However, in the vast majority of metrics, especially in the ability to recognize lesion targets, the key dice similarity coefficient (DSC)/F1 metrics and intersection over union (IoU) metrics, etc., the proposed UltraLight VM-UNet is leading. Further, Figure 4 shows the comparison between the proposed method and the comparison method in terms of inference speed and memory usage. As can be seen from the figure, the inference speed of the proposed UltraLight VM-UNet maintains an efficient value, which is faster than any other equivalent Mamba-based lightweight model. In terms of memory usage, the proposed UltraLight VM-UNet has the lowest memory usage (batch size uniformly 8) of all the compared models, which again demonstrates the excellent trade-off between lightweight and performance of UltraLight VM-UNet.

**Table 1:** Experimental comparisons of the proposed UltraLight VM-UNet with the best lightweight and classical models

Model	Parameters (millions)	GFLOPs	DSC/F1	SE/Recall	SP	ACC	IoU	Prec
**Comparison experiments on the ISIC2017 dataset**								

U-Net^21^	2.009	3.224	0.8989	0.8793	0.9812	0.9613	0.8165	0.9196
SCR-Net^22^	0.801	1.567	0.8898	0.8497	0.9853	0.9588	0.8015	0.9340
Swin-Unet^23^	27.176	7.724	0.8670	0.8427	0.9754	0.9494	0.7652	0.8928
ATTENTION SWIN U-NET^8^	46.910	14.181	0.8859	0.8492	0.9847	0.9591	0.7998	0.9444
C^2^SDG^24^	22.001	7.972	0.8938	0.8859	0.9765	0.9588	0.8081	0.9019
VM-UNet^25^	27.427	4.112	0.9070	0.8837	0.9842	0.9645	0.8298	0.9302
VM-UNet v2^26^	22.771	4.400	0.9045	0.8768	0.9849	0.9637	0.8256	0.9168
MALUNet^18^	0.175	0.083	0.8896	0.8824	0.9762	0.9583	0.8008	0.9295
LightM-UNet^19^	0.403	0.391	0.9080	0.8839	0.9846	0.9649^a^	0.8303	0.9321
EGE-UNet^10^	0.053	0.072	0.9073	0.8931	0.9816	0.9642	0.8302	0.9219
DermoSegDiff^27^	45.112	38.636	0.8789	0.8435	0.9815	0.9545	0.7841	0.9174
LiteMamba-Bound^28^	0.957	1.591	0.9054	0.8743	0.9861^a^	0.9643	0.8272	0.9374
UltraLight VM-UNet (Our)	0.049^a^	0.060^a^	0.9091^a^	0.9053^a^	0.9790	0.9646	0.8334^a^	0.9481^a^

**Comparison experiments on the ISIC2018 dataset**								

U-Net^21^	2.009	3.224	0.8851	0.8735	0.9744	0.9539	0.7938	0.8970
SCR-Net^22^	0.801	1.567	0.8886	0.8892	0.9714	0.9547	0.7995	0.8880
Swin-Unet^23^	27.176	7.724	0.8342	0.8142	0.9648	0.9343	0.7155	0.8551
ATTENTION SWIN U-NET^8^	46.910	14.181	0.8540	0.8057	0.9826^a^	0.9480	0.7683	0.9183
C^2^SDG^24^	22.001	7.972	0.8806	0.8970	0.9643	0.9506	0.7867	0.8648
VM-UNet^25^	27.427	4.112	0.8891	0.8809	0.9743	0.9554	0.8004	0.8966
VM-UNet v2^26^	22.771	4.400	0.8902	0.8959	0.9702	0.9551	0.8020	0.8672
MALUNet^18^	0.175	0.083	0.8931	0.8890	0.9725	0.9548	0.8028	0.8827
LightM-UNet^19^	0.403	0.391	0.8898	0.8829	0.9765	0.9555	0.8013	0.8902
EGE-UNet^10^	0.053	0.072	0.8819	0.9009^a^	0.9638	0.9510	0.7887	0.8637
DermoSegDiff^27^	45.112	38.636	0.8672	0.8344	0.9693	0.9421	0.7697	0.8760
LiteMamba-Bound^28^	0.957	1.591	0.8929	0.8911	0.9714	0.9546	0.8019	0.8894
UltraLight VM-UNet (Our)	0.049^a^	0.060^a^	0.8940^a^	0.8680	0.9781	0.9558^a^	0.8056^a^	0.9197^a^

Comparison experiments on the ${\text{PH}}^{2}$ dataset								

U-Net^21^	2.009	3.224	0.9060	0.9255	0.9440	0.9381	0.8282	0.8874
SCR-Net^22^	0.801	1.567	0.8989	0.9114	0.9446	0.9339	0.8164	0.8868
Swin-Unet^23^	27.176	7.724	0.8631	0.8613	0.9359	0.9119	0.7591	0.8649
ATTENTION SWIN U-NET^8^	46.910	14.181	0.8850	0.8886	0.9363	0.9213	0.7990	0.8838
C^2^SDG^24^	22.001	7.972	0.9030	0.9137	0.9476	0.9367	0.8231	0.8925
VM-UNet^25^	27.427	4.112	0.9033	0.9131	0.9483	0.9369	0.8237	0.8948
VM-UNet v2^26^	22.771	4.400	0.9050	0.9160	0.9485	0.9380	0.8265	0.8797
MALUNet^18^	0.175	0.083	0.8865	0.8922	0.9425	0.9263	0.8010	0.8993
LightM-UNet^19^	0.403	0.391	0.9156	0.9129	0.9613^a^	0.9457	0.8443	0.9103
EGE-UNet^10^	0.053	0.072	0.9086	0.9198	0.9502	0.9404	0.8325	0.8978
DermoSegDiff^27^	45.112	38.636	0.8928	0.8779	0.9577	0.9320	0.8064	0.9082
LiteMamba-Bound^28^	0.957	1.591	0.9217	0.9341	0.9558	0.9488	0.8548	0.9097
UltraLight VM-UNet (Our)	0.049^a^	0.060^a^	0.9265^a^	0.9345^a^	0.9606	0.9521^a^	0.8631^a^	0.9187^a^

a These values represent the best performance.

**Figure 5:**
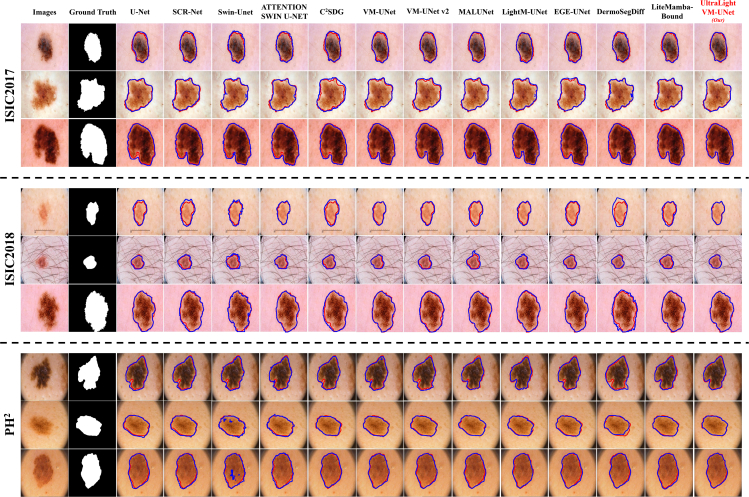
Visualization of segmentation graphs for comparison experiments of three publicly available skin lesion segmentation datasets The red contour line is the true value and the blue contour line is the predicted value.

To more directly represent the competitive nature of UltraLight VM-UNet in terms of segmentation performance, we visualized the segmentation results (Figure 5). In addition, we have also visualized the segmentation results of several state-of-the-art lightweight and classical medical image segmentation models. From the visualizations, it can be concluded that the segmentation results of UltraLight VM-UNet have smooth, clear, and more accurate boundaries. This shows that UltraLight VM-UNet not only outperforms the rest of the current lightweight models in terms of parameters and computational complexity, but also remains competitive in terms of performance.

## Discussion

### Ablation experiments

#### VM with different levels of parallelism

**Figure 6:**
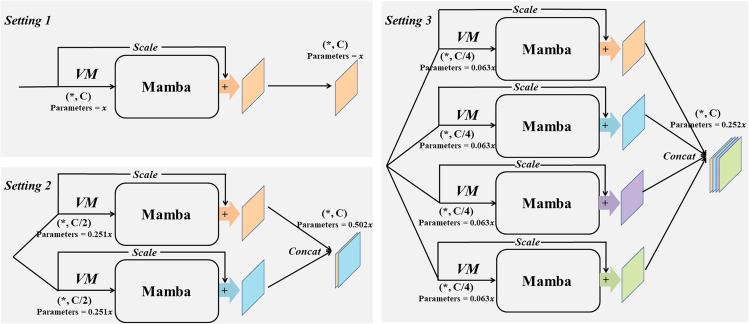
Settings for ablation experiments with Vision Mamba used in different parallel ways (PVM Layer)

**Figure 7:**
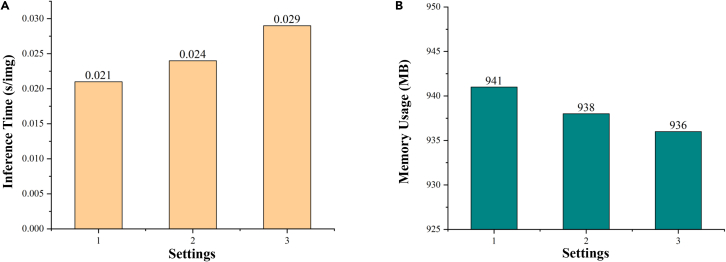
Comparison of inference time and memory usage of Vision Mamba with different parallel connections (A) Comparison of inference time of Vision Mamba with different parallel connections. (B) Comparison of memory usage of Vision Mamba with different parallel connections.

To verify the validity of the proposed method of VM with different parallelism, we performed a series of ablation experiments. As shown in Figure 6, we performed three different settings. Setting 1 is a conventional connection of VM, setting 2 is a connection using a parallel connection of two VMs with half the number of channels, and setting 3 is a connection using parallel connection of four VMs each with C/4 the number of channels. By analyzing the parameters of Mamba in "mamba parameter impact analysis," assuming that the parameter of this module is x for setting 1 of the traditional VM connection method, setting 2 can be calculated with a parameter of 0.502x and setting 3 with a parameter of 0.252x. Table 2 shows the results of this ablation experiment, and it should be noted that the parameters here refer to the parameters of the overall model (which contains the Conv Block and the skip-connection part). The parameters of setting 2 and setting 3 are 51.47% and 36.03%, respectively, of the parameters of setting 1 for the traditional VM connection method, while the GFLOPs as a whole do not change much. In terms of performance, the lowest parameter of setting 3 still maintains better segmentation performance. This is due to the focus on the target area by multiple PVM, but this also affects the ability of the model to recognize the background, which results in the SP index for setting 3 being the lowest. However, in the vast majority of the indices, especially the contrast lesion target recognition ability, i.e., the key DSC/F1 index and the IoU index are leading. Furthermore, in Figure 7, which shows the inference speed and memory usage for three different parallel settings, it can be concluded that, due to the design of the ultra-lightweight architecture, although parallel processing increases the number of matrix operations slightly, the difference between four-parallel operations is only slightly manifested at the millisecond level at most. However, as can be seen from Table 2, quad-parallel processing will reduce the number of parameters by a significant 74.8%. Therefore, to realize the ultra-lightweight architecture design with millisecond difference and excellent performance, we adopt setting 3 as the key structure of the proposed PVM Layer.

**Table 2:** Ablation experiments on the effect of Vision Mamba in different parallel connections

Settings	Parameters (millions)	GFLOPs	DSC/F1	SE/Recall	SP	ACC	IoU	Prec
1	0.136	0.060	0.9069	0.8861	0.9834	0.9644	0.8266	0.9354
2	0.070	0.060	0.9073	0.8866	0.9835^a^	0.9645	0.8284	0.9398
3	0.049^a^	0.060^a^	0.9091^a^	0.9053^a^	0.9790	0.9646^a^	0.8334^a^	0.9481^a^

a These values represent the best performance.

#### Parallelization of different SSM variants

In the methods, we detail the key to the influence of the parameters of Mamba, represented by SSM. However, many current studies propose improvements based on SSM to adapt it to 2D image processing. In Liu et al.,^11^ researchers proposed SS2D for visual image processing. In Ruan andXiang,^25^ researchers proposed VM-UNet for medical image segmentation by combining SS2D with UNet framework. In addition, based on SS2D, Wu et al.^29^ proposed high-order SS2D (H-SS2D) for medical image segmentation. The parameter and performance effects of SS2D and H-SS2D using parallel approach are shown in Table 3. From the table, it is concluded that the parameters and GFLOPs of (*P*) SS2D are reduced by 81.35% and 61.97%, respectively, while those of (*P*) H-SS2D are reduced by 80.34% and 57.14%, respectively. The above results reveal that the parallel approach is effective in reducing parameters and GFLOPs not only for Mamba represented by SSM but also for different variants of SSM at the same time.

**Table 3:** Impact of adopting parallelism for different SSM variants

Methods	SSM variants	Params	GFLOPs	DSC	SE	SP	ACC	IoU	Prec
“1” VM-UNet	SS2D	27.427M	4.112	0.9070	0.8837	0.9842	0.9645	0.8298	0.9302
“2” VM-UNet	SS2D	27.427M	4.112	0.8891	0.8809	0.9743	0.9554	0.8004	0.8966
“3” VM-UNet	SS2D	27.427M	4.112	0.9033	0.9131	0.9483	0.9369	0.8237	0.8948
“1” VM-UNet	(*P*) SS2D	5.116M	1.564	0.9159	0.8843	0.9886	0.9682	0.8366	0.9352
“2” VM-UNet	(*P*) SS2D	5.116M	1.564	0.8977	0.8996	0.9734	0.9584	0.8009	0.9002
“3” VM-UNet	(*P*) SS2D	5.116M	1.564	0.9185	0.9340	0.9525	0.9465	0.8377	0.9034
“1” H-vmunet	H-SS2D	8.973M	0.742	0.9172	0.9056	0.9831	0.9680	0.8471	0.9291
“2” H-vmunet	H-SS2D	8.973M	0.742	0.8966	0.8952	0.9741	0.9581	0.8073	0.8756
“3” H-vmunet	H-SS2D	8.973M	0.742	0.9146	0.9295	0.9509	0.9440	0.8427	0.9002
“1” H-vmunet	(*P*) H-SS2D	1.764M	0.318	0.9066	0.8825	0.9843	0.9644	0.8344	0.9349
“2” H-vmunet	(*P*) H-SS2D	1.764M	0.318	0.8948	0.9067	0.9695	0.9567	0.8079	0.8744
“3” H-vmunet	(*P*) H-SS2D	1.764M	0.318	0.9181	0.9254	0.9569	0.9467	0.8473	0.9020

(*P*) shows the adoption of quadruple parallelism to replace the original form; “1” indicates experiments on the ISIC2017 dataset, “2” indicates the ISIC2018 dataset, and “3” indicates the ${\text{PH}}^{2}$ dataset.

#### Plug-and-play PVM Layer

The proposed PVM Layer can simply replace the base building blocks of any model, which include but are not limited to Convolution, Vision Transformers, Mamba, VM, and so on. Table 4 shows the powerful plug-and-play capabilities of the PVM Layer. From the table, it can be concluded that, after replacing the base building blocks or key functional modules of any model with PVM Layer, there is a significant decrease in parameters and GFLOPs, and the performance is still clearly competitive. In addition, MALUNet and EGE-UNet, as the most advanced lightweight models, after replacing the key modules in the PVM Layer, the parameters and GFLOPs can still be effectively reduced, and the performance is improved. With the above results, it is shown that the powerful plug-and-play feature of the PVM Layer is used to significantly reduce the parameters and GFLOPs of arbitrary models.

**Table 4:** Comparative experiments where the PVM Layer directly replaces modules from different models, with results including parameters, computational complexity, and performance

Methods	Parameters and computational complexity				Performance evaluation					
	Params	$D^{P}$(%)	GFLOPs	$D^{G\;}$(%)	DSC/F1	SE/Recall	SP	ACC	IoU	Prec
UNet^21^	2.009M	80.38	3.224	78.74	0.8989	0.8793	0.9812	0.9613	0.8165	0.9196
UNet_*Conv*	0.394M		0.686		0.8974	0.8667	0.9842	0.9612	0.8147	0.9211
Att UNet^30^	3.581M	45.10	8.575	29.61	0.8821	0.8423	0.9836	0.9560	0.7891	0.9259
Att UNet_*Conv_block*	1.966M		6.036		0.8857	0.8414	0.9857	0.9575	0.7921	0.9241
SCR-Net^22^	0.812M	81.90	1.567	72.75	0.8898	0.8497	0.9853	0.9588	0.8015	0.9340
SCR-Net_*Conv*	0.147M		0.427		0.9058	0.8847	0.9833	0.9640	0.8233	0.9370
Swin-UNet^23^	27.176M	76.11	7.724	75.48	0.8670	0.8427	0.9754	0.9494	0.7652	0.8928
Swin-UNet_*Vision Transformer*	6.491M		1.894		0.8734	0.8445	0.9783	0.9521	0.7710	0.8953
META-Unet^31^	22.209M	94.77	5.140	91.91	0.9068	0.8801	0.9836	0.9639	0.8301	0.9327
META-Unet_*ResNet Layer*	1.161M		0.416		0.9047	0.8915	0.9807	0.9633	0.8264	0.9285
MHorUNet^3^	9.585M	90.35	0.864	81.94	0.9132	0.8974	0.9834	0.9666	0.8348	0.9289
MHorUNet_*Horblock*	0.925M		0.156		0.9107	0.8806	0.9870	0.9662	0.8318	0.9300
HSH-UNet^9^	18.803M	84.64	9.362	90.58	0.9108	0.8907	0.9864	0.9654	0.8214	0.9356
HSH-UNet_*HSHB*	2.888M		0.882		0.9001	0.8938	0.9776	0.9612	0.8161	0.9286
MALUNet^18^	0.175M	26.86	0.083	10.84	0.8896	0.8824	0.9762	0.9583	0.8008	0.9295
MALUNet_*DGA*	0.128M		0.074		0.9025	0.8623	0.9882	0.9635	0.8234	0.9327
EGE-UNet^10^	0.053M	1.89	0.072	1.39	0.9073	0.8931	0.9816	0.9642	0.8302	0.9219
EGE-UNet_*GHPA*	0.052M		0.071		0.9092	0.8941	0.9823	0.9650	0.8332	0.9241
VM-UNet^25^	27.427M	60.94	4.112	56.10	0.9070	0.8837	0.9842	0.9645	0.8298	0.9302
VM-UNet_*Vision Mamba*	10.713M		1.805		0.8885	0.8517	0.9841	0.9582	0.8120	0.9177
VM-UNet v2^26^	22.771M	73.69	4.400	65.43	0.9045	0.8768	0.9849	0.9637	0.8256	0.9168
VM-UNet v2_*Vision Mamba*	5.991M		1.521		0.8916	0.8692	0.9804	0.9586	0.8205	0.9119
LightM-UNet^19^	0.403M	72.95	0.391	0.26	0.9080	0.8839	0.9846	0.9649	0.8303	0.9321
LightM-UNet_*Mamba Layer*	0.109M		0.390		0.9045	0.8668	0.9878	0.9642	0.8292	0.9297

Italics in the method column (e.g., *_Conv*) indicate that the PVM Layer replaces the convolution module. In particular, we replace modules containing Convolution, Vision Transformer, Mamba, and Vision Mamba, covering the four most commonly used base modules. $D^{P}$ and $D^{G\;}$ denote the percentage decrease in parameters and GFLOPs, respectively, after replacing with PVM Layer.

#### Impact of components in the UltraLight VM-UNet

A series of ablation experiments were performed to verify the impact of each module in the UltraLight VM-UNet. As shown in Table 5, we replace the PVM Layer of the encoder, decoder, respectively, with a standard convolution with convolution kernel 3. In addition, we also replace the PVM Layer of the encoder and decoder at the same time with a standard convolution. Also, Baseline_SCABnotapplicable indicates that the skip-connection part of UltraLight VM-UNet does not use the SAB and CAB modules. From the table, we can conclude that, by replacing the PVM Layer of the encoder and decoder separately, the parameters increased by 63.26% and the GFLOPs increased in both, while the performance decreased in both. In particular, after replacing the PVM Layer of the encoder and decoder simultaneously, the parameters increase by 151% and the GFOLPs increase by 25%. In summary, it is shown that, after replacing the PVM Layer, there is a decrease in all performance aspects and an increase in both parameters and GFLOPs. This again proves the crucial role of PVM Layer. What is more, although the parameters and GFOLPs were further reduced with the removal of the SAB and CAB modules. However, the performance also exhibits some degradation, which is due to the ability of the SAB and CAB modules to combine multi-scale feature information for learning, which improves the sensitivity to lesions and accelerates the model convergence.^18^ Therefore, to balance the performance and computational complexity relationship, UltraLight VM-UNet employs the SAB and CAB modules as a bridge for skip connections to further improve segmentation performance. Even so, the performance, parameters, and GFLOPs of UltraLight VM-UNet are still in the forefront compared with the rest of the state-of-the-art lightweight models available currently.

**Table 5:** Ablation experiments on the effect of each module in the UltraLight VM-UNet

Methods	Params	GFLOPs	DSC/F1	SE/Recall	SP	ACC	IoU	Prec
UltraLight VM-UNet (baseline)	0.049M	0.060	0.9091^a^	0.9053^a^	0.9790	0.9646^a^	0.8334^a^	0.9481^a^
Baseline_Encoder_Conv	0.080M	0.071	0.9033	0.8643	0.9880	0.9638	0.8237	0.9461
Baseline_Decoder_Conv	0.080M	0.064	0.8958	0.8512	0.9881^a^	0.9612	0.8113	0.9454
Baseline_(En+De)_Conv	0.123M	0.075	0.9065	0.8784	0.9855	0.9645	0.8291	0.9365
Baseline_SCAB not applicable	0.033M^a^	0.058^a^	0.9029	0.8767	0.9841	0.9631	0.8221	0.9436

a These values represent the best performance.

#### Impact of different channel numbers in UltraLight VM-UNet

In addition, to verify the impact of different channel numbers in the UltraLight VM-UNet, we conducted a series of ablation experiments. As shown in Table 6, we set up four different combinations of channel numbers, including [8,16,24,32,48,64], [8,16,32,64,128,256], [16,32,64,128,256,512], and [32,64,128,256,512,1024], respectively. In addition, we conducted experiments both in the parallel-free manner (labeled by “1”) and in the parallel manner (labeled by “2”). From the table, it can be concluded that the parameters and GFLOPs at [8,16,24,32,48,64] are the lowest, while the overall difference in performance is not very large. However, as the number of channels increases, both parameters and GFLOPs show a significant rise. The parameter increases from 0.136M to 13.607M for the group without parallelism and from 0.049M to 3.305M for the group with parallelism. In particular, the average decrease in parameters for the same number of channel combinations using the parallel approach over the non-parallel approach is 72.70%, which is extremely close to the percentage of decrease in the four-parallel approach over the non-parallel approach analyzed in the methods section (74.80%). In addition, at [8,16,24,32,48,64], the parameters are more susceptible to the rest of the modules, so the decrease is not as large as for the rest of the channel number combinations. However, its overall parameters and GFLOPs reach an impressive 0.049M and 0.060, and show strong competitive segmentation performance.

**Table 6:** Ablation experiments on the effect of combining different channel numbers in the UltraLight VM-UNet

Methods	Params	$D^{P}\left(74.80\%\right)$	GFLOPs	DSC/F1	SE/Recall	SP	ACC	IoU	Prec
“1” [8,16,24,32,48,64]	0.136M	63.98%^(−10.82%)^	0.060	0.9069	0.8861	0.9834	0.9644	0.8266	0.9354
“2” [8,16,24,32,48,64]	0.049M		0.060	0.9091	0.9053	0.9790	0.9646	0.8334	0.9481
“1” [8,16,32,64,128,256]	0.909M	75.47%^(+0.67%)^	0.074	0.9018	0.8749	0.9840	0.9627	0.8279	0.9417
“2” [8,16,32,64,128,256]	0.223M		0.074	0.9079	0.8965	0.9809	0.9644	0.8282	0.9326
“1” [16,32,64,128,256,512]	3.479M	75.63%^(+0.83%)^	0.259	0.9049	0.8981	0.9789	0.9630	0.8311	0.9402
“2” [16,32,64,128,256,512]	0.848M		0.259	0.9056	0.8906	0.9815	0.9637	0.8224	0.9450
“1” [32,64,128,256,512,1024]	13.607M	75.71%^(+0.91%)^	0.970	0.9053	0.8878	0.9821	0.9637	0.8214	0.9459
“2” [32,64,128,256,512,1024]	3.305M		0.970	0.9012	0.8812	0.9819	0.9622	0.8297	0.9334

$D^{P}$ denotes the percentage of parameter reduction with the parallel approach; 74.80% denotes the theoretical percentage reduction of localized module parameters derived from the Mamba analysis in the methods; “1” indicates non-parallel connection method and “2” indicates a quadruple parallel connection method.

### Discussion in practical engineering

**Figure 8:**
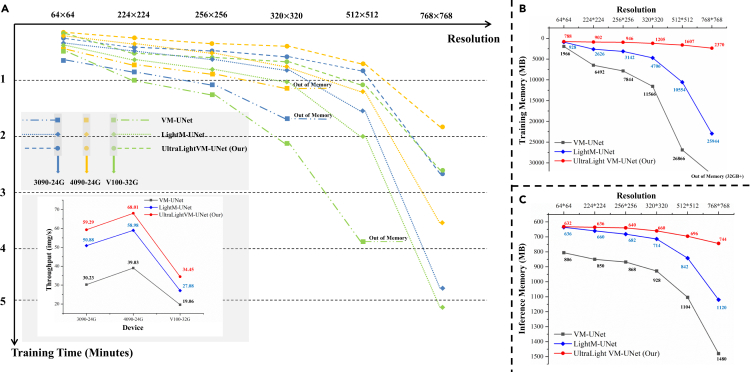
Discussion of the results in practical engineering applications (A) Comparison of training speed and inference throughput of the proposed model with baseline model and the lightest Mamba-based medical image segmentation model at different resolutions and different devices. (B) Comparison of the memory required for training the proposed model with the baseline model and the lightest Mamba-based medical image segmentation model at different resolutions. (C) Comparison of the memory required for inference of the proposed model with the baseline model and the lightest Mamba-based medical image segmentation model at different resolutions.

In practical engineering applications, it is common for different devices to be used for training and inference in skin lesion segmentation tasks. To further discuss the superiority of the proposed method, we provide the throughput of our model versus the baseline model on different devices in Figure 8. Specifically, the throughput of our proposed model on 3090, 4090, and V100 is about two times higher than that of the baseline model (VM-UNet). In particular, compared with LightM-UNet, our model achieves a 20%-40% improvement in throughput across a variety of hardware platforms.

In addition, the need for segmentation of skin lesion images at different resolutions is also encountered through practical engineering applications. Based on this problem, we tested the training speed and inference speed (throughput) of the proposed model versus the baseline model in Figure 8 at different resolutions. However, a batch size of 1 is used for inference, so the inference speed (throughput) does not change much at different resolutions. In particular, the advantage of our model is shown more clearly on the high resolution 512×512. Nonetheless, researchers prefer to use 256×256 resolution in processing images of skin lesion.^8^^,^^9^^,^^10^^,^^18^^,^^29^

### Discussion under the task of non-skin lesion segmentation

To further validate the potential of the proposed UltraLight VM-UNet, we conducted comprehensive experiments on two additional datasets: the Autooral dataset^32^ and the Spleen dataset.^33^ These datasets comprise oral ulcer (cancer) images acquired through endoscopy and spleen images in CT mode, respectively. The Autooral dataset, developed by Jiang et al.,^32^ contains high-quality oral ulcer (cancer) images obtained by oral endoscopy. We maintained experimental consistency with the original study’s protocols. The Spleen dataset, established by Memorial Sloan Kettering Cancer Center,^33^ provides CT modality data for spleen segmentation, and the experimental configuration adhered to the methodology outlined by Wu et al.^29^ to ensure a fair comparison. The results of the experiments on the Autooral dataset and the Spleen dataset are presented in Table 7. Since our focus is on the exploration of lightweight, we performed experiments with the current state-of-the-art lightweight and classical medical image segmentation models. From the table, it can be concluded that although the UltraLight VM-UNet is slightly inferior to other models in terms of DSC and sensitivity (SE) metrics on endoscopic mode and CT mode; however, it still shows strong competitiveness in terms of SP and accuracy (ACC) metrics. In particular, compared with other current state-of-the-art lightweight models (MALUNet, LightM-UNet, and EGE-UNet), we lead in several metrics. The UltraLight VM-UNet demonstrates competitiveness through experiments in both endoscopic and CT modalities, as well as the dermoscopic segmentation task that is the focus of this paper. In particular, the UltraLight VM-UNet is more competitive for dermatoscope segmentation tasks.

**Table 7:** Experimental results with state-of-the-art lightweight and classical medical image segmentation models on Autooral dataset and Spleen dataset

Model	Parameters (millions)	GFLOPs	DSC/F1	SE/Recall	SP	ACC	IoU	Prec
**Comparison experiments on the Autooral dataset (non-skin lesion segmentation task)**								

U-Net^21^	2.009	3.224	0.7480^b^	0.7282	0.9815	0.9617	0.5575	0.7290
SCR-Net^22^	0.801	1.567	0.7069	0.6148	0.9896	0.9602	0.5467	0.8315^a^
Swin-Unet^23^	27.176	7.724	0.4484	0.3675	0.9770	0.9295	0.2890	0.5750
ATTENTION SWIN U-NET^8^	46.910	14.181	0.6463	0.5032	0.9954^a^	0.9571	0.4774	0.7732
C^2^SDG^24^	22.001	7.972	0.7210	0.6554	0.9862	0.9604	0.5638	0.8012^b^
VM-UNet^25^	27.427	4.112	0.7639^a^	0.7555^a^	0.9811	0.9636^a^	0.5970^b^	0.6879
VM-UNet v2^26^	22.771	4.400	0.7461	0.7285^b^	0.9813	0.9602	0.5580	0.7060
MALUNet^18^	0.175	0.083	0.6318	0.6500	0.9655	0.9409	0.4832	0.7201
LightM-UNet^19^	0.403	0.391	0.6551	0.6130	0.9781	0.9497	0.4483	0.5831
EGE-UNet^10^	0.053^b^	0.072^b^	0.5893	0.7263	0.9375	0.9211	0.4178	0.4958
DermoSegDiff^27^	45.112	38.636	0.3569	0.2565	0.9845	0.9338	0.2134	0.7599
LiteMamba-Bound^28^	0.957	1.591	0.7425	0.7166	0.9833	0.9609	0.5981^a^	0.7533
UltraLight VM-UNet (Our)	0.049^a^	0.060^a^	0.7255	0.6637	0.9903^b^	0.9621^b^	0.5742	0.7673

**Comparison experiments on the Spleen dataset (non-skin lesion segmentation task)**								

U-Net^21^	2.010	5.037	0.9441^b^	0.9604^a^	0.9989	0.9983^b^	0.8679	0.9283
SCR-Net^22^	0.812	2.449	0.9181	0.9122	0.9988	0.9976	0.8486	0.9220
Swin-Unet^23^	27.193	12.084	0.6758	0.5526	0.9923	0.9818	0.4832	0.5541
ATTENTION SWIN U-NET^8^	46.945	22.175	0.7829	0.6662	0.9994^a^	0.9945	0.6433	0.9341
C^2^SDG^24^	22.010	12.457	0.9354	0.9263	0.9991^b^	0.9981	0.8787	0.9448^a^
VM-UNet^25^	27.428	6.425	0.9418	0.9429	0.9991^b^	0.9982	0.8859	0.9248
VM-UNet v2^26^	22.771	6.874	0.9445^a^	0.9315	0.9990	0.9982	0.8902^a^	0.9419
MALUNet^18^	0.178	0.130	0.9310	0.9305	0.9989	0.9979	0.8709	0.9315
LightM-UNet^19^	0.405	0.587	0.9329	0.9459	0.9988	0.9982	0.8646	0.9317
EGE-UNet^10^	0.053^b^	0.113^b^	0.8992	0.8866	0.9987	0.9975	0.8251	0.9347
DermoSegDiff^27^	45.112	60.368	0.9266	0.9507^b^	0.9987	0.9979	0.8777	0.9196
LiteMamba-Bound^28^	0.957	2.484	0.9214	0.9455	0.9990	0.9981	0.8897^b^	0.9314
UltraLight VM-UNet (Our)	0.049^a^	0.094^a^	0.9168	0.8991	0.9991^b^	0.9984^a^	0.8566	0.9430^b^

a These values represent the best performance.

b These values represent the second-best performance.

### Conclusion

In this study, we conduct a detailed analysis of the key factors influencing parameter efficiency in Mamba. Building upon these insights, we propose a PVM Layer for efficient deep feature processing. The PVM Layer consists of four VM modules operating in parallel, with each module responsible for processing one-quarter of the original input channels. This is due to the fact that the number of input channels to the SSM in Mamba has an explosive effect on the number of parameters, and the VM parameters for processing a quarter of the number of channels are 6.3% of the original VM parameters, which is an explosive reduction of 93.7%. In addition, the PVM Layer can be generalized to any Mamba variant (P x M Layer). Based on the PVM Layer, we propose the UltraLight VM-UNet with a parameter of only 0.049M and GFLOPs of only 0.060. The UltraLight VM-UNet parameters are 99.82% lower than those of the traditionally pure VM-UNet model and 87.84% lower than those of the lightest LightM-UNet model. In addition, we experimentally demonstrated on three publicly available skin lesion datasets that the UltraLight VM-UNet has equally strong performance competitiveness with such low parameters. We also discuss the performance of the UltraLight VM-UNet for non-skin lesion segmentation tasks and demonstrate its potential competitiveness.

In particular, it is important to note that in this work we have not just proposed a lightweight model but an in-depth exploration of Mamba for lightweight research in healthcare settings. Based on this work, Mamba may become a new mainstream module for lightweight model.

## Methods

### Related work

Medical image segmentation, as one of the important branches in image segmentation, is also one of the research directions to which many researchers have devoted their efforts. Among them, multi-scale variation problem and feature refinement learning are the key problems in medical image segmentation.^34^ Also, skin lesion segmentation has rich and varied feature information with regard to the high lethality caused by malignant melanoma,^35^ which has led many researchers to carry out a series of studies around skin lesion segmentation.^3^^,^^8^^,^^9^

Medical image segmentation algorithms represented by skin lesions have been rapidly developed after the advent of U-Net. In Aghdam et al.,^8^ an inhibition operation of the attention mechanism in cascade operation has been proposed for skin lesion segmentation based on Swin-Unet.^23^ The MHorUNet^3^ model proposes a high-order spatial interaction UNet model for skin lesion segmentation. In Wu et al.,^9^ an adaptive selection of a higher-order UNet model for order interaction has been proposed for skin lesion segmentation. DSU-Net^36^ proposes to utilize convolutional neural networks and transformers to construct a bipolar UNet for skin lesion segmentation, which proceeds from coarse to fine through two stages. However, the joint use of convolutional neural networks and transformers further makes the architecture more bloated. BDFormer^37^ proposes a new boundary-aware bi-decoder transformer, which employs a single-encoder and bi-decoder framework for skin lesion segmentation and expanding boundary segmentation. DermoSegDiff^27^ proposes to introduce denoised diffusion probabilistic models into the skin lesion segmentation task which effectively integrates noise and semantic information, and proposes a loss function adapted to the boundary region to improve lesion boundary recognition. EGE-UNet^10^ proposes a lightweight model for skin lesion segmentation that employs Group multi-axis Hadamard Product Attention module and Group Aggregation Bridge module, which significantly reduces the number of parameters and computation while maintaining high performance.

MALUNet^18^ is another lightweight model for skin lesion segmentation proposed by researchers, which introduces multiple attention mechanism modules to significantly reduce the number of parameters and computational complexity while maintaining high performance in skin lesion segmentation tasks. In addition, there are many algorithms based on U-Net improved for skin lesion segmentation. However, it is common for researchers to add richer modules to the model to improve the accuracy of recognition, but this also significantly increases the parameters and computational complexity of the model. After the emergence of VM, LightM-UNet^19^ was proposed to reduce the number of parameters in the model based on Mamba. LightM-UNet further extracts the deep semantics and tele-relationships by using the residual VM, and achieves better performance with a smaller number of parameters. In addition, U-Mamba^38^ introduced VM into the U-framework, but its having a large number of parameters (173.53M) limits its use in real clinical settings. LiteMamba-Bound^28^ proposed a lightweight model designed for skin lesion segmentation task, which improves skin lesion recognition by introducing a channel-attentive bi-Mamba module and an inverse-attentive boundary module.

In this paper, to solve the current problem of large model parameters and to reveal the key factors affecting Mamba parameters. We propose UltraLight VM-UNet based on Mamba with a parameter of only 0.049M. In addition, we propose a PVM method for processing deep features, named the PVM Layer, based on a detailed theoretical analysis. It is a plug-and-play module that can simply replace the convolutional layers and transformers to significantly reduce the number of model parameters and maintain excellent segmentation performance.

### Datasets

To validate that the proposed UltraLight VM-UNet maintains competitive performance with only 0.049M parameters, we conducted experiments on three publicly available dermatologic lesion datasets. The ISIC2017^39^ and ISIC2018^40^ datasets are two large datasets published by the International Skin Imaging Collaboration (ISIC), respectively. The ${\text{PH}}^{2}$^41^ dataset is a small skin lesion dataset, so we used ${\text{PH}}^{2}$ as an external validation using training weights from ISIC2017.

For the ISIC2017 dataset we acquired 2,000 images as well as dermatoscope images with segmentation mask labels. Among them, the dataset was randomly divided, 1,250 images were used for model training, 150 images were used for model validation, and 600 images were used for model testing. The initial size of the images is 576×767 pixels, and we standardize the size to 256×256 pixels when inputting the model.

For the ISIC2018 dataset we acquired 2,594 images as well as dermatoscope images with segmentation mask labels. Among them, the dataset was randomly divided, 1,815 images were used for model training, 259 images were used for model validation, and 520 images were used for model testing. The initial size of the images is 2,016×3,024 pixels, and we standardize the size to 256×256 pixels when inputting the model.

For the ${\text{PH}}^{2}$ dataset we acquired 200 images as well as dermatoscope images with segmentation mask labels. All 200 images were used for external validation. The initial size of the images was 768×560 pixels and we standardized the size to 256×256 pixels.

### Implementation details

The proposed UltraLight VM-UNet is shown in Figure 2. The experiments were all implemented based on Python 3.8 and Pytorch 1.13.0. A single NVIDIA V100 GPU with 32 GB of memory was used for all experiments. All experiments used the same data augmentation operations to more fairly determine the performance of the model, including horizontal and vertical flips, and random rotation operations. BceDice loss function was used, with AdamW^42^ as the optimizer, a training epoch of 250, a batch size of 8, and a cosine annealing learning rate scheduler with an initial learning rate of 0.001 and a minimum learning rate set to 0.00001.

### Evaluation metrics

The DSC, SE, SP, ACC, IoU, and precision (Prec) are the most commonly used evaluation metrics in medical image segmentation. The DSC is used to measure the similarity between the ground truth and the predicted segmentation maps, and it is equivalent to the F1 score in image segmentation tasks. SE is primarily used to measure the percentage of true positives among true positives and false negatives, it is equivalent to Recall. SP is mainly used to measure the percentage of true negatives among true negatives and false positives. ACC is mainly used to measure the proportion of correctly classified samples. IoU is primarily used to measure the degree of overlap between the predicted segmentation region and the true segmentation region. Prec is mainly used to measure the proportion of correctly segmented pixels in the predicted segmentation region.

(Equation 15) $$\text{DSC}/F1=\frac{2\text{TP}}{2\text{TP}+\text{FP}+\text{FN}}$$

(Equation 16) $$\text{ACC}=\frac{\text{TP}+\text{TN}}{\text{TP}+\text{TN}+\text{FP}+\text{FN}}$$

(Equation 17) $$\text{SE}/\text{Recall}=\frac{\text{TP}}{\text{TP}+\text{FN}}$$

(Equation 18) $$\text{SP}=\frac{\text{TN}}{\text{TN}+\text{FP}}$$

(Equation 19) $$\text{IoU}=\frac{\text{TP}}{\text{TP}+\text{FP}+\text{FN}}$$

(Equation 20) $$\text{Prec}=\frac{\text{TP}}{\text{TP}+\text{FP}}$$

where TP denotes true positive, TN denotes true negative, FP denotes false positive, and FN denotes false negative.

## Resource availability

### Lead contact

Requests for further information and resources should be directed to and will be fulfilled by the lead contact, Qing Chang (robie0510@hotmail.com).

### Materials availability

This study did not generate new materials.

### Data and code availability

Our source code is available at GitHub (https://github.com/wurenkai/UltraLight-VM-UNet) and has been archived at Zenodo.^43^ The ISIC2017 dataset is from Codella et al.^39^ and is available at https://challenge.isic-archive.com/data/#2017. The ISIC2018 dataset is from Codella et al.^40^ and is available at https://challenge.isic-archive.com/data/#2018. The ${\text{PH}}^{2}$ dataset is from Mendonça et al.^41^ and is available at https://drive.google.com/file/d/1AEMJKAiORlrwdDi37dRqbqXi6zLmnU3Q/view?usp=sharing.

## Acknowledgments

This work was supported partly by Medicine-engineering Interdisciplinary Project set up by 10.13039/501100008050University of Shanghai for Science and Technology (2023-LXY-RUIJIN01Z).

## Author contributions

Conceptualization, R.W. and Y.L.; methodology, R.W. and Y.L.; writing – original draft, R.W.; writing – review & editing, R.W., Y.L., G.N., and P.L.; formal analysis, R.W. and G.M.; validation, Y.L. and P.L.; project administration, Q.C.; funding acquisition, Q.C.; **s**upervision, Q.C.

## Declaration of interests

The authors declare no competing interests.
